# Cardiac shockwave therapy in patients with chronic refractory angina pectoris

**DOI:** 10.1007/s12471-016-0821-y

**Published:** 2016-03-02

**Authors:** J. Vainer, J. H. M. Habets, S. Schalla, A. H. P. Lousberg, C. D. J. M. de Pont, S. A. Vöö, B. T. Brans, J. C. A. Hoorntje, J. Waltenberger

**Affiliations:** Department of Cardiology, Maastricht University Medical Centre, Maastricht, The Netherlands; Department of Nuclear Medicine, Maastricht University Medical Centre, Maastricht, The Netherlands; Department of Cardiovascular Medicine, University Hospital Muenster, Muenster, Germany

**Keywords:** Angina, stable, Myocardial ischaemia, Radionuclide imaging, Echocardiography, Shock Waves, Ultrasonic

## Abstract

**Background:**

Cardiac shockwave therapy (CSWT) might improve symptoms and decrease ischaemia burden by stimulating collateral growth in chronic ischaemic myocardium. This prospective study was performed to evaluate the feasibility and safety of CSWT.

**Methods:**

We included 33 patients (mean age 70 ± 7 years, mean left ventricular ejection fraction 55 ± 12 %) with end-stage coronary artery disease, chronic angina pectoris and reversible ischaemia on myocardial scintigraphy. CSWT was applied to the ischaemic zones (3–7 spots/session, 100 impulses/spot, 0.09 mJ/mm^2^) in an echocardiography-guided and ECG-triggered fashion. The protocol included a total of 9 treatment sessions (3 treatment sessions within 1 week at baseline, and after 1 and 2 months). Clinical assessment was performed using exercise testing, angina score (CCS class), nitrate use, myocardial scintigraphy, and cardiac magnetic resonance (CMR) 1 and 4 months after the last treatment session.

**Results:**

One and 4 months after CSWT, sublingual nitrate use decreased from 10/week to 2/week (*p* < 0.01) and the angina symptoms diminished from CCS class III to CCS class II (*p* < 0.01). This clinical improvement was accompanied by an improved myocardial uptake on stress myocardial scintigraphy (54.2 ± 7.7 % to 56.4 ± 9.4 %, *p* = 0.016) and by increased exercise tolerance at 4-month follow-up (from 7.4 ± 2.8 to 8.8 ± 3.6 min *p* = 0.015). No clinically relevant side effects were observed.

**Conclusion:**

CSWT improved symptoms and reduced ischaemia burden in patients with end-stage coronary artery disease without relevant side effects. The study provides a solid basis for a randomised multicentre trial to establish CSWT as a new treatment option in end-stage coronary artery disease.

## Introduction

Despite advances in medical and invasive revascularisation therapy a substantial number of patients show progression to end-stage ischaemic coronary artery disease with chronic refractory angina symptoms and no further revascularisation options [1]. Long-term mortality in patients with refractory angina who are not candidates for revascularisation is low. Over 70 % of patients with refractory angina survive 9 years from the time of diagnosis. Therapeutic options for this growing population should therefore focus on chest pain relief and improved quality of life [2]. For these ‘no-option patients’ [3], novel therapeutic strategies have been developed to improve both quality of life and prognosis, which include enhanced external counter-pulsation [4], spinal cord stimulation [5], therapeutic angiogenesis [6], coronary sinus intervention [7] and newer drugs [1]. Cardiac shockwave therapy (CSWT) is a novel treatment strategy targeting angiogenesis with concomitant improvement of the local microcirculation.

A shockwave is a single pressure pulse with a short (< 1 msec) positive spike with an amplitude up to 100 MPa followed by a lower amplitude tensile part lasting several microseconds [8]. The highly localised physical forces of shockwaves are used to disintegrate urolithiasis in urology [9] and, with lower shockwave energy, in orthopaedics to induce neovascularisation, and improve local perfusion and tissue regeneration [10, 11]. Clinical studies with statistically significant positive outcomes have been performed for urolithiasis [9], calcifying tendinitis [10], plantar fasciitis [12], epicondylitis of the lateral humerus [11], delayed healing of bone fractures [13], and diabetic and vascular ulcers [14, 15].

In predominantly animal model studies, the following effects of shockwave therapy have been observed: Cavitation (rapid formation and collapse of vapour pockets in and outside cells with the formation of jet streams and free radicals) [16], thermal and chemical effects [17], molecular biological and cellular changes [13], and hyperstimulation analgesia [18]. These effects result in complex changes in tissue such as the formation of new bone, neo-angiogenesis and nerve stimulation.

Pre-clinical studies and limited clinical data suggest a positive effect of very low energy shockwaves (0.09 mJ/mm^2^) on myocardial ischaemia and left ventricular function [19–29]. Upregulation of vascular endothelial growth factor (VEGF) mRNA and protein expression are likely to be responsible for the increasing capillary density observed in ischaemic myocardium treated by shockwaves [20].

The current study was performed to evaluate the feasibility and safety of CSWT in patients with chronic refractory angina pectoris and to provide initial insight into the persistence of any clinical effects during mid-term follow-up of up to 4 months.

## Methods

The study was approved by the Maastricht University Medical Centre Ethics Committee and written informed consent was obtained from all participants.

### Definition of refractory angina

Refractory angina pectoris is a chronic condition characterised by the presence of angina caused by coronary insufficiency in the presence of coronary artery disease, which cannot be controlled by a combination of medical therapy, angioplasty and coronary bypass surgery. The presence of reversible myocardial ischaemia should be clinically established to be the cause of the symptoms. Chronic is defined as a duration of more than 3 months [30].

### Shockwave therapy

During a 4-year period, 55 patients with chronic refractory angina and end-stage coronary artery disease were referred to our institution. Twenty-two patients were excluded, mostly because of absence of reversible ischaemia or unstable symptoms. Thirty-three patients fulfilled the inclusion criteria (reversible ischaemia on myocardial perfusion scintigraphy (SPECT), stable ischaemia-related symptoms for more than 3 months prior to the CSWT initiation without a change in medication) and were included and treated with shockwave therapy [20] after further revascularisation options had been evaluated for each patient and individually found to be non-viable by the Heart Team of our institution.

**Fig. 1:**
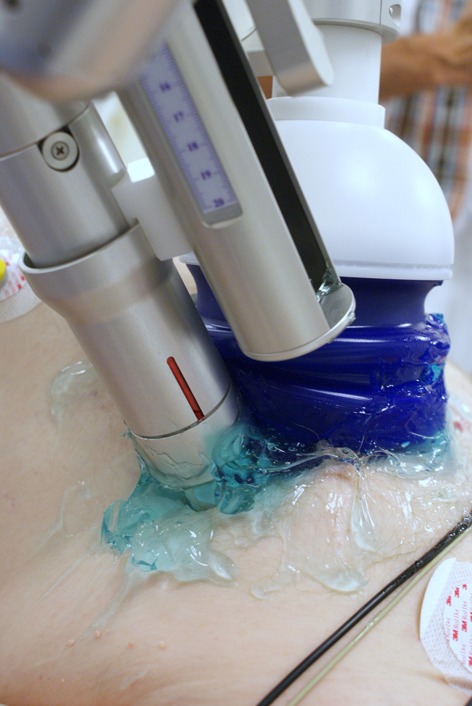
The shockwave applicator mounted in a holder together with a standard echocardiography transducer. Echocardiography is used to locate predefined ischaemic regions and to guide the focus of the shockwave applicator

The outpatient shockwave treatment protocol included 3 treatments per week at 0, 1 and 2 months resulting in a total of 9 treatment sessions. Per session, 3–7 different spots were treated. With the patient in a supine position, CSWT was applied to the ischaemic zones (100 impulses/spot, energy flux 0.09 mJ/mm^2^ using the Cardiospec^TM^ device (Medispec Ltd, Germantown, USA) in an echocardiography-guided and ECG-triggered fashion. Briefly, the Cardiospec^TM^ device creates shockwaves using an electrohydraulic method. An electric discharge within a water filled applicator evaporates a small portion of water that generates shockwaves. The shockwaves are reflected and focused, with a focus size of 8 mm width and 25 mm depth, by means of an applicator to the zone of myocardial ischaemia. The ischaemic regions were detected by SPECT and during treatment located by ultrasound using a transthoracic echocardiography transducer mounted on a special holder on the shockwave applicator (Fig. 1).

Clinical assessment of the patients was performed by exercise testing (modified Bruce protocol), angina score (CCS class), nitrate use, echocardiography and SPECT at baseline (prior to first treatment session), and 1 month and 4 months after the last treatment session. Cardiac magnetic resonance imaging (CMR) was performed for safety reasons in the first 8 patients at baseline, 1 and 4 months.

The safety of the shockwave therapy was assessed with continuous rhythm monitoring as well as blood pressure measurements during the intervention. Further, repeated electrocardiograms and echocardiography during and after the treatment were performed. Blood samples were taken to exclude potential myocardial damage. The clinical status of the patient was assessed and the skin controlled for burns or erythema immediately and 1 h after the procedure.

### Echocardiography

Before therapy, and 1 and 4 months after shockwave therapy, a complete echocardiographic examination was performed to evaluate left ventricular systolic and diastolic function and size, segmental wall motion and valve function using Sonos 5500 ultrasound systems with S3-1 (1–3 MHz) transthoracic transducers or IE33 ultrasound systems with S5-1 (1–5 Mhz) transducers (Philips Medical Systems, Andover, MA, USA). In addition, image quality in supine position was evaluated since shockwave therapy can only be performed in the supine position. During treatment, the ischaemic zones were placed in the middle of the sector scan.

As described above, diagnostic echocardiography was also performed during shockwave therapy to steer the shockwave beam to the myocardial regions of interest, as previously defined by SPECT.

### Myocardial perfusion imaging

SPECT was performed by a one-day protocol, using either Tl-201 (111 MBq) or Tc-99 m-sestamibi (270 MBq after stress, 1000 MBq at rest) [31]. Per patient, the same stress protocol was used in all 3 tests (modified Bruce treadmill exercise, 0.5 mg/kg body weight persantin IV, or both). Acquisition was performed using 60 or 64 projections over 180º (RAO 45º to LPO 45º) with non-circular orbit (Philips Skylight dual-headed gamma-camera, Philips Medical Systems, Andover, MA, USA). Acquisition time was 30 s per projection during stress acquisition and 20 s during rest acquisition. Reconstruction into tomographic trans-axial images was done using filtered back projection with no scatter or attenuation correction applied. Analysis of perfusion images utilised the Quantitative Perfusion SPECT (QPS) software (QPS, Cedar-Sinai, Los Angeles, CA) installed with the custom-developed normal database. An experienced technologist performed processing, while the semi-quantitative visual judgment was made by the consensus of two experienced observers, a nuclear cardiologist (SV) or an experienced nuclear medicine physician (BB or CdP), blinded to the clinical data. The visual interpretation of perfusion images was based on short-axis and vertical and horizontal long-axis tomograms [32] divided into a 17-segment model, according to the American Heart Association [33]. Supporting the visual interpretation, a semi-quantitative analysis was also performed: each segment was scored automatically based on a 5-point scoring system (0, normal uptake; 1, mild decreased uptake; 2, moderately decreased uptake; 3, severely decreased uptake; 4, absence of uptake), as previously described [34, 35]. The sum of the stress scores of all segments (SSS) and the sum of the rest scores of all segments (SRS) were determined from these values. A summed difference score (SDS) was calculated as the difference between SSS and SRS. SDS > 1 was defined as reversible perfusion defect indicating ischaemia.

**Fig. 2:**
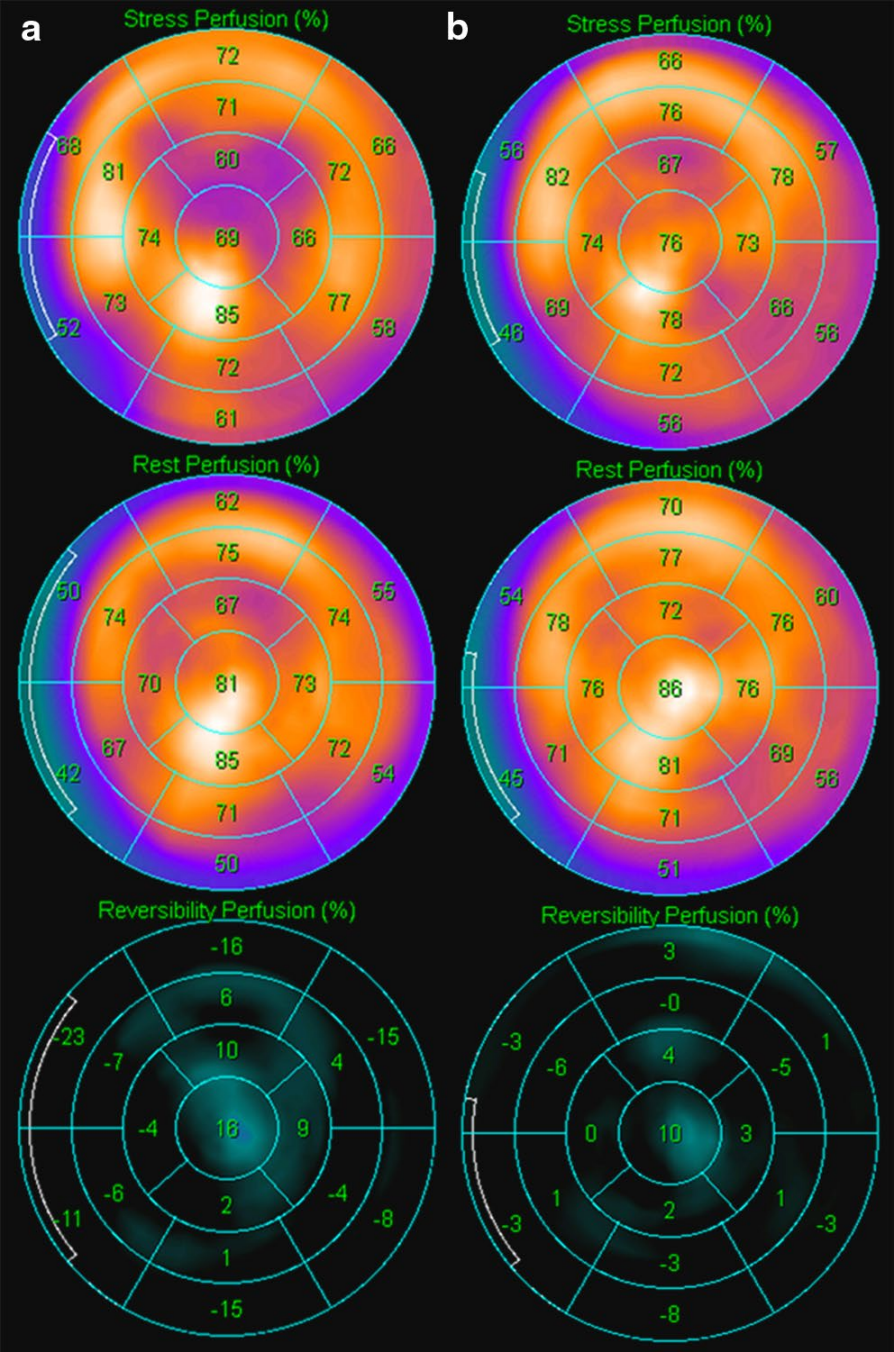
Quantitative polar map (17 segments) displaying the myocardial perfusion as the raw counts based on myocardial scintigraphy analysis. The pixel with the maximum raw count is set to the maximum colour scale brightness and corresponds to a value of 100. The numbers correspond with the average ‘raw’ pixel value in the related segments

Scintigraphy results were divided into two categories: (*i*) negative, defined as having homogenous radioactive distributions in myocardium, on both stress and rest scans, and SDS = 0; (*ii*) positive, defined as reversible and fixed perfusion defects. Reversible perfusion defects were considered the segments with a decreased tracer uptake at stress but with partial improvement or complete normalisation of tracer uptake at rest. Fixed pattern showed that localised segments of decreased perfusion were unchanged between the stress and rest images. Reversible perfusion defects with SDS > 1 were considered to represent myocardial ischaemia, whereas fixed perfusion defects were considered to be myocardial scar. The inter-observer variability was found to be < 10 %. For evaluation of the effect of CSWT quantitative polar maps were constructed of the stress and rest perfusion, using the Autoquant/QPS software package (Philips Medical Systems, Andover, MA, USA). This map displays the myocardial perfusion as raw counts. The pixel with the maximum raw count is set to the maximum colour scale brightness and corresponds to a value of 100. Values of the segmental counts corresponded to the average pixel value in that segment. Values of corresponding segments between stress and rest images were compared, stratifying between treated and untreated segments (Fig. 2).

### Cardiac magnetic resonance

Patients were examined with a 1.5 T MR scanner (Gyroscan Intera, Philips Medical Systems, Best, the Netherlands) as previously described [36]. Cine images were acquired for functional analysis using a steady-state free precession sequence (TR/TE 3.8/1.9 ms, flip angle 50^°^) in 2-, 3- and 4-chamber long axis and contiguous short axis views. Standard late gadolinium enhancement images were acquired to evaluate the presence of myocardial infarction (breath hold multislice T1-weighted 3D inversion-recovery gradient-echo sequence, slice thickness 12 mm, gap − 6 mm, TR/TE 4.2/1.3 ms, flip angle 15^°^, FOV 400 mm, resolution 256 × 256) 10 min after intravenous administration of 0.2 mmol/kg Gadolinium-DTPA (Magnevist, Bayer Schering AG, Berlin, Germany). Inversion times were adjusted to null normal myocardium (200–300 ms).

CMR images were analysed with the CAAS MRV 3.0 software (Pie Medical Imaging, Maastricht, the Netherlands). Areas of myocardial infarction were visually assessed on late enhancement images. If present, areas were quantified by planimetry using a signal cut-off of > 2 SD above the mean signal intensity of remote myocardium in the same slice and expressed as a percentage of total LV mass.

### Clinical chemistry analysis

Blood samples were taken after the second treatment session from all patients and the following laboratory parameters were analysed to detect or exclude myocardial damage as a potential side effect of CSWT: serum creatinine kinase, (high sensitive) troponin T, aspartate aminotransferase and alanine aminotransferase.

### Statistical methods

Statistical analysis was performed using SPSS software (SPSS, Inc., Chicago, IL, USA). Continuous variables with no/mild skew were presented as mean ± SD, skewed measures as median. Discrete variables were summarised as frequencies and percentages. Differences were assessed using a paired sample t-test for normally distributed data or Wilcoxon signed rank two related sampled analyses for other distributions.

## Results

The study group consisted of 33 patients (27 males, mean age 69.7 years). The baseline characteristics of the patient population are summarised in Table 1. The treatment duration was 28 ± 8 min (mean ± SD) per session depending on the acoustic window, heart rate and number of spots treated (3–7 spots per session).

**Table 1:** Baseline characteristics of treated patients (*n* = 33)

Age (years; mean, SD)	69.7 ± 8
Male	27 (82 %)
Body mass index (mean, SD)	29 ± 4.6
Heart rate (beats/min; mean, SD)	67 ± 11
Systolic blood pressure (mmHg; mean, SD)	132 ± 19
Diastolic blood pressure (mmHg; mean, SD)	77 ± 10
Smoking	8 (24 %)
Positive family history for coronary disease	11 (33 %)
Diabetes mellitus	15 (46 %)
Hypertension	25 (76 %)
Hypercholesterolaemia	31 (94 %)
Chronic obstructive pulmonary disease	4 (12 %)
Three-vessel disease	28 (85 %)
Two-vessel disease	5 (15 %)
History of PCI	18 (55 %)
History of multiple PCIs	12 (36 %)
History of CABG	25 (76 %)
History of repeated CABG	6 (18 %)
History of stroke	5 (15 %)
Peripheral vascular disease	8 (24 %)
Aspirin	21 (64 %)
Clopidogrel	10 (30 %)
Oral anticoagulation	8 (24 %)
Nitrates	30 (91 %)
Βeta-blockers	28 (85 %)
Calcium antagonists	26 (79 %)
ACE inhibitor	12 (36 %)
AT II antagonist	7 (21 %)
Insulin	6 (18 %)
Oral antidiabetic agents	10 (30 %)

CSWT was well tolerated without major side effects. Transient dizziness shortly after the first treatment session was reported from 4 patients. Creatinine kinase (106 ± 52 U/l) or TnT levels were not elevated following the treatment sessions in any of the patients. The electrocardiograms did not show any significant changes after treatment. No arrhythmias were observed and no clinical signs of dyspnoea or coughing as a sign of lung contusion were noticed. No skin erythema or burns were observed. There were no changes in medication during the treatment and the follow-up.

### Clinical benefit of CSWT

Following CSWT, the clinical status improved significantly: The use of sublingual nitrates decreased from a median of 10 per week (range 1–25) pre-CSWT to 2 per week (range 0–20) at 1 month and to 2 per week (range 0–7) at 4 months post-CSWT (*p* < 0.01). Simultaneously, angina complaints decreased by at least one CCS class in 25 patients at 1 month and in all but two patients at 4 months after CSWT (*p* < 0.01) (Table 2). The exercise tolerance slightly improved during follow-up (from 7.4 ± 2.8 to 8.3 ± 3.9 and to 8.8 ± 3.6 min, *p* = 0.015).

**Table 2:** Effect of cardiac shockwave therapy in 33 patients with end-stage ischaemic heart disease treated with cardiac shock-wave therapy

	Baseline	One-month follow-up	Four-month follow-up
Angina CCS class	3.0 ± 0.3	1.9 ± 0.6; *p* < 0.01	1.7 ± 0.7; *p* < 0.01
Nitrate use/week	9.7 ± 7.0	2.2 ± 3.9; *p* < 0.01	1.5 ± 1.9; *p* < 0.01
Exercise test (minutes)	7.4 ± 2.8	8. 3 ± 3.9; *p* = NS	8.8 ± 3.6; *p* = 0.015
Nuclear perfusion stress treated segments (%)	54.2 ± 7.7	55.7 ± 6.5; *p* = 0.037	56.4 ± 9.4; *p* = 0.016
Nuclear perfusion stress untreated segments (%)	59.0 ± 7.7	59.5 ± 6.3; *p* = NS	59.4 ± 6.7; *p* = NS
Nuclear perfusion rest treated segments (%)	60.4 ± 8.4	60.3 ± 7.1; *p* = NS	60.3 ± 9.3; *p* = NS
Nuclear perfusion rest untreated segments (%)	60.9 ± 6.7	60.7 ± 6.3; *p* = NS	60.8 ± 7.2; *p* = NS
Ejection fraction (%)	53.2 ± 12.3	53.7 ± 12.1; *p* = NS	
Scar (CMR) % (8 pts)	5.3 ± 4.3	5.6 ± 4.3; *p* = NS	5.8 ± 5.0; *p* = NS

Data are presented as mean ± standard deviation (SD), statistical significance compared with the baseline.

### Clinical benefit of CSWT correlates with improved regional myocardial perfusion

**Fig. 3:**
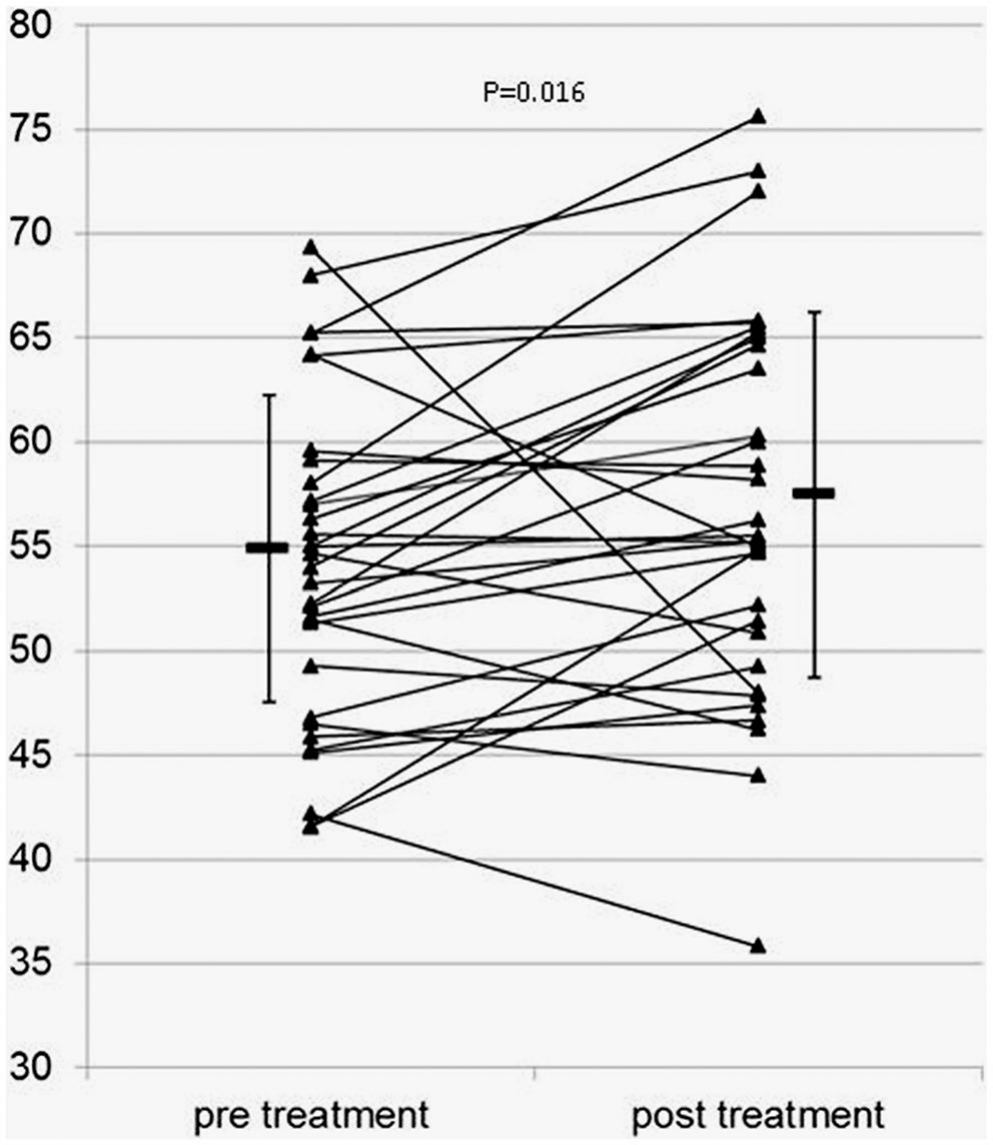
Perfusion scintigraphy at stress; comparison 4 month post CSWT with baseline (prior to CSWT). X axis: baseline and 4-month follow–up. Y axis: relative percentage of perfusion of treated segment

Regional myocardial perfusion revealed a significant decrease of ischaemia burden in treated regions following CSWT demonstrated by SPECT on stress images after 1 month (*p* = 0.037) and 4 months (*p* = 0.016) (Table 2, Fig. 3). As expected, myocardial perfusion at rest was unaltered in treated and untreated areas before and after CSWT. Also, left ventricular ejection fraction was not found to be different before and after the shockwave treatment. We investigated a subgroup of 8 patients for scar formation using gadolinium delayed enhancement CMR. This substudy did not reveal a change in scar tissue volume following CSWT (Table 2).

## Discussion

The main finding of our study is that CSWT therapy is capable of improving ischaemic symptoms and reducing ischaemia burden in patients with end-stage coronary artery disease, the so called ‘no-option patients’. The beneficial effects were observed in the absence of detectable relevant side effects. Our study demonstrates an improvement of the clinical status of the patients undergoing CSWT.

Moreover, our study provides mechanistic insight into the improvement of regional myocardial perfusion following CSWT, and thereby provides a solid basis for the increased exercise tolerance of these patients during follow-up of up to 4 months. The SPECT data suggest that at least part of the positive effect might be due to improved perfusion in the treated ischaemic zones at stress. Consistent with this observation and as expected, no changes in resting perfusion were detectable in treated and untreated myocardial areas nor in the stress perfusion of untreated areas.

These results confirm the preliminary findings of Fukumoto et al., who investigated 9 patients with end-stage coronary artery disease [19]. The limited improvement of stress perfusion by about 2 % for the whole group is in agreement with the proposed mechanism of angiogenesis on the micro-vascular level. This positive effect on myocardial perfusion was also found by Kaller et al. [28] and is in line with the results of Vasyuk et al. on a hibernating myocardium and ischaemic heart failure after shock wave treatment [22]. The improvement of left ventricular systolic function found in their study and in the study by Zuoziene et al. [26] was not confirmed by our results and results of Alluni et al. [27] and Kaller et al. [28]. The near-normal left ventricular ejection fraction at baseline in our study and the study of Alluni et al. [27] and Kaller et al. [28] might be responsible for this discrepancy.

The treatment was well tolerated and not accompanied by any serious side effects known from studies on shockwave therapy in lithotripsy [37] or orthopaedic indications [10]. This is in agreement with findings of others [19, 22, 23]. Shockwaves with low energy flux (0.09 mJ/mm^2^) seem to be safe [20, 22, 23, 26, 29].

## Limitations

The limited sample size and the lack of a control group are the main limitations of this study. A ‘regression to the mean’ phenomenon [38] as an explanation of diminished symptoms and improved perfusion cannot be excluded; however, the stable symptoms for more than 3 months prior to treatment initiation without a change in medication (run-in phase) strongly suggest a real therapeutic effect. Progression of coronary artery disease may reduce the positive effect of shockwave treatment. Long-term follow-up is needed to shed more light on the fate of these patients.

## Conclusions

Decreased ischaemic burden in about two-thirds of the patients combined with alleviation of angina symptoms makes CSWT an interesting alternative in the difficult to treat group of patients with refractory angina pectoris. The main advantage of this relatively time-consuming outpatient-based method is its non-invasive character in combination with no significant side-effects. A further validation in a properly sized randomised placebo-controlled trial is needed before CSWT can be introduced into clinical routine.

### Funding

This work was supported in part by a ‘profileringsfonds’ of the Maastricht University Medical Centre (to J.V. and J.W.).

### Conflict of interest

None declared.
